# Increasing mapping precision of genome-wide association studies: to genotype and impute, sequence, or both?

**DOI:** 10.1186/s13059-017-1255-6

**Published:** 2017-06-19

**Authors:** Zhaoming Wang, Nilanjan Chatterjee

**Affiliations:** 1 Department of Computational Biology, St. Jude Children’s Research Hospital, Memphis, Tennessee USA; 2 Department of Biostatistics, Bloomberg School of Public Health and Department of Oncology, School of Medicine, Johns Hopkins University, Baltimore, Maryland USA

## Abstract

Fine-mapping to identify causal variants in genome-wide association studies remains challenging. A recent study provides guidance for future research.

## Introduction

Genome-wide association studies (GWAS) search for marker variants indirectly associated with certain diseases and/or traits. They assume that markers are in linkage disequilibrium (LD) with underlying causal variants. Compared to the initial discovery of associations, the fine-mapping effort required to identify causal variants—whether statistical or functional—remains challenging in this post-GWAS era.

Reference panels such as those from the HapMap and 1000 Genome projects have improved, with better genome coverage including tens of millions of catalogued variants. Availability of these resources has led to methods for genotype imputation, in which genotypes for all variants in the reference are statistically inferred. Subsequent association analysis on imputed variants might allow refinement of the association hits originally discovered through array-based GWAS. However, fine-mapping through imputation is limited by the poor accuracy of imputed genotypes for rare variants, and the existence of underlying rare causal variants in reference panels cannot be guaranteed.

Theoretically, with the application of whole-genome sequencing (WGS) in GWAS, all variants—including underlying causal variants—can be directly genotyped and tested to achieve the simultaneous goal of both discovery and fine-mapping. However, it is expensive to perform WGS on large numbers of samples, so it is unlikely to be adopted as a main approach for GWAS anytime soon. A key question is, what is the best strategy to increase mapping precision: to genotype and impute, sequence, or both?

In a recent elegant paper, Wu et al. [1] attempted to statistically quantify the mapping precision of GWAS imputation and WGS through simulation experiments based on empirical WGS data from 3642 individuals who took part in the 1000 UK Genomes study. Their findings provide guidance for future study designs and suggest that alternative ways of mapping the common and rare causal variants underlying GWAS associations should be sought.

## Rejecting the synthetic association hypothesis

In the “synthetic association” hypothesis, the association underlying a common variant is driven by many rare causal variants residing in a neighboring genomic region in LD with one particular allele of the common variant [2]. However, the authors showed that the causal variants underlying associations detected through common variants, which comprise the majority of loci discovered by GWAS to date, are generally also common. This finding concurs with those of many targeted re-sequencing studies, which have been largely unsuccessful in identifying rare and functional variants in GWAS-associated loci. One important caveat to note, however, is the authors’ presumption that only one causal variant exists in their simulation analysis, whether rare or common.

## Precision of fine-mapping approaches

The authors measured the proportion of GWAS hits expected within a given physical distance from selected causal variants. They did this by simulating and comparing three typical study designs involving single nucleotide polymorphism (SNP) microarray genotyping, followed by imputation (into HapMap2, the 1000 Genomes Project Phase 1, and 1000 Genomes Project Phase 3 (1KGP3)), as well as the WGS-based approach. For the three imputation-based strategies, over 94% of GWAS hits fall within 100 kb of causal variants with a minor allele frequency >0.01. The proportion increased slightly to 98% with the WGS-based approach. The authors deduced that GWAS followed by imputation has comparable precision to WGS, and the latter is cost-ineffective for fine-mapping common variants.

However, for rare variants, mapping precision for the best imputed dataset using 1KGP3 as a reference was substantially lower than that for WGS. Simulation studies showed that 98% of WGS-based GWAS hits fell within 100 kb of the causal variants with a minor allele frequency <0.01, whereas only 68% met the criteria for 1KGP3-based imputation. Underlying this finding is the fact that most of the rare variants in the 1000 UK Genomes study were not present in the imputation reference set. A limited number of LD surrogates also exist within a small genomic region harboring each rare causal variant.

## Genome coverage versus sample size

The authors noted that genome coverage is more important for fine-mapping precision than the sample size of the imputation reference set. However, the latter is important for imputation accuracy, and thus the statistical power, in detecting associations for rare variants. Particularly for rare variants, power loss caused by imputation is similar to sample size reduction and should therefore affect the fine-mapping precision. A possible explanation for the lack of observation of any remarkable effect of the sample size of the imputation reference set is that the simulated effect sizes were large. Thus, the power for detecting underlying associations was sufficiently high.

Researchers are now shifting from imputation based on 1KGP3, which includes about 5000 haplotypes, to the new Haplotype Reference Panel, which includes about 65,000 haplotypes [3]. The increase in sample size and coverage will surely improve imputation accuracy for lower allele frequency spectra, and thus the ability to fine-map array-based GWAS for rare causal variants.

## The case of multiple causal variants

The authors acknowledged that a weakness of their paper is their failure to consider loci with multiple causal variants, which may underlie some disease associations. For example, the best-known loci conferring germline cancer susceptibility are 8q24 and 5p15.3, which both include multiple independent signals and are associated with several cancers. A fine-mapping study of 5p15.33 revealed at least six independent associations with five different cancers [4]. When modeling multiple rare casual variants, it may be important to apply burden or aggregated tests in which the number of mutant alleles within a gene or genomic region is counted for association analysis. This would obtain better power to detect associations compared to single variant tests. However, investigation of the likely causal roles of individual rare variants is not likely to be straightforward.

## What is on the horizon?

Decreasing costs will make WGS-based GWAS for large sample numbers more feasible. In the meantime, meta-analyses based on imputation are being put to good use to combine new and existing array-based GWAS studies, including fine-mapping efforts. For example, using this strategy, rare variants of moderately large effects in *BRCA2* and *CHEK2* genes have been associated with lung cancer risk [5]. To take advantage of such a strategy, international consortia have come together to design custom arrays and conduct another wave of GWAS discoveries through genotyping and imputation. One such effort is the design of OncoArray [6]; this comprises a genome-wide backbone that tags most common genetic variants, and variants for fine-mapping in established cancer susceptibility loci, including rare variants derived from sequencing studies. OncoArray has already been used to genotype more than 450,000 samples around the world. Nevertheless, imputation-based approaches remain limited. A WGS-based approach can overcome these limitations, and will become the mainstream for rare variant association studies in the near future.

Whether or not it is an advantage to employ WGS in GWAS depends on the allelic spectrum or genetic architecture of the disease/trait under investigation. For example, a recent WGS-based GWAS for type 2 diabetes [7] found variants associated with the disease to be overwhelmingly common, and that most fell within regions previously discovered by SNP array-based GWAS. On the other hand, a WGS-based GWAS for amyotrophic lateral sclerosis [8] simultaneously detected and fine-mapped a novel locus containing a rare functional variant; heritability analysis indicated a disproportionate contribution of low-frequency SNPs to disease predisposition.

An important consideration for the future is that rare variants, which are mostly in weak LD with neighboring variants, increase the number of independent tests, and thus the multiple-testing burden to control for false negative signals. In light of this, Wu et al. recommend applying a more stringent threshold of 5 × 10^−9^. Furthermore, functional annotations such as epigenetic footprints, transcriptional factor binding motifs, and expression quantitative trait loci could be used to improve power to detect associations. For example, a weighted Bonferroni adjustment based on the enrichment of sequence annotations among association signals might be used [9].

Rare variants, even if—in total—they contribute substantially to heritability, are likely to be distributed over many thousands of loci, each with small effects [10]. Thus, ultimately, the sample size for WGS needs to be very large, possibly in the tens of thousands to hundreds of thousands, to make a comparable number of discoveries to those we have seen for array-based GWAS. Large-scale international consortia are needed to combine genetic data with full genome coverage (i.e., WGS) to increase discovery power and fine-mapping precision to gain further insights into the biological mechanisms underlying complex diseases and traits.

## Abbreviations

1KGP3: 1000 Genomes Project Phase 3
GWAS: Genome-wide association study
LD: Linkage disequilibrium
SNP: Single nucleotide polymorphism
WGS: Whole genome sequencing
